# Assessing the availability of LLINs for continuous distribution through routine antenatal care and the Expanded Programme on Immunizations in sub-Saharan Africa

**DOI:** 10.1186/s12936-016-1309-3

**Published:** 2016-05-04

**Authors:** Katherine Theiss-Nyland, Michael Lynch, Jo Lines

**Affiliations:** Infectious Disease Epidemiology Department, London School of Hygiene and Tropical Medicine, Keppel Street, London, UK; Global Malaria Programme, World Health Organization, Geneva, Switzerland; Malaria Branch, Division of Parasitic Diseases and Malaria, Centers for Disease Control and Prevention, Atlanta, GA USA; Disease Control Department, London School of Hygiene and Tropical Medicine, Keppel Street, London, UK

## Abstract

**Background:**

In addition to mass distribution campaigns, the World Health Organization (WHO) recommends the continuous distribution of long-lasting insecticidal nets (LLINs) to all pregnant women attending antenatal care (ANC) and all infants attending the Expanded Programme on Immunization (EPI) services in countries implementing mosquito nets for malaria control. Countries report LLIN distribution data to the WHO annually. For this analysis, these data were used to assess policy and practice in implementing these recommendations and to compare the numbers of LLINs available through ANC and EPI services with the numbers of women and children attending these services.

**Methods:**

For each reporting country in sub-Saharan Africa, the presence of a reported policy for LLIN distribution through ANC and EPI was reviewed. Prior to inclusion in the analysis the completeness of data was assessed in terms of the numbers of LLINs distributed through all channels (campaigns, EPI, ANC, other). For each country with adequate data, the numbers of LLINs reportedly distributed by national programmes to ANC was compared to the number of women reportedly attending ANC at least once; the ratio between these two numbers was used as an indicator of LLIN availability at ANC services. The same calculations were repeated for LLINs distributed through EPI to produce the corresponding LLIN availability through this distribution channel.

**Results:**

Among 48 malaria-endemic countries in Africa, 33 malaria programmes reported adopting policies of ANC-based continuous distribution of LLINs, and 25 reported adopting policies of EPI-based distribution. Over a 3-year period through 2012, distribution through ANC accounted for 9 % of LLINs distributed, and LLINs distributed through EPI accounted for 4 %. The LLIN availability ratios achieved were 55 % through ANC and 34 % through EPI. For 38 country programmes reporting on LLIN distribution, data to calculate LLIN availability through ANC and EPI was available for 17 and 16, respectively.

**Conclusions:**

These continuous LLIN distribution channels appear to be under-utilized, especially EPI-based distribution. However, quality data from more countries are needed for consistent and reliable programme performance monitoring. A greater focus on routine data collection, monitoring and reporting on LLINs distributed through both ANC and EPI can provide insight into both strengths and weaknesses of continuous distribution, and improve the effectiveness of these delivery channels.

## Background

Long-lasting insecticidal nets (LLINs) have been the mainstay of vector control for malaria prevention. The World Health Organization (WHO) recommends universal coverage of LLINs, defined as one LLIN for every two people within a household, for malaria-endemic countries and regions [1]. Eighty-eight countries, 39 in Africa, distribute LLINs free of charge [2]. The main channel for LLIN distribution since the early 2000s has been mass campaigns. Since 2007, WHO has also recommended the continuous distribution of LLINs to all pregnant women through routine antenatal care (ANC) and to all children under 1 year through the Expanded Programme on Immunizations (EPI), to complement campaigns, and maintain coverage of the most biologically vulnerable people during the intervals between mass campaigns [3–6].

The routine health services of ANC and EPI have important advantages as LLIN distribution points because their target populations are especially vulnerable to malaria, and because, compared to other health services, they tend to achieve relatively high and equitable levels of access for these target groups in most countries. Globally, approximately 83 % of women receive ANC at least once during their pregnancy [7], and 85 % of children complete their vaccination schedule [8]. In 41 of 45 countries in the WHO African Region, the policy is to distribute LLINs free of charge [9]. In countries with an ANC distribution policy, the first ANC visit is generally used as the point-of-contact for LLIN distribution, in line with the WHO recommendation [6]. There is no specific WHO recommendation as to when LLINs should be distributed in EPI [4, 6], and a wide range of time-points is used in practice, from birth (with BCG vaccine for tuberculosis) to 9 months of age (with measles vaccine). Diphtheria-tetnus-pertussus-1 (DTP1) vaccination at 6 weeks of age is the most common distribution point [6].

Each year, to prepare for the production of the World Malaria Report, national malaria programmes in endemic countries provide WHO with data on the adoption of LLIN distribution policies and the total LLINs made available for distribution through all channels (campaign, ANC, EPI, other) [2, 10]. These data have not previously been used to assess the extent to which ANC and EPI distribution channels have been utilized for LLINs in practice. The WHO has collected reports of the numbers of nets distributed since 2000 at country level, and in this analysis national programme reports of the nets distributed through ANC or EPI were compared with reports of the total number of women attending ANC or children attending EPI, to calculate an LLIN availability ratio for each channel.

## Methods

The LLIN continuous distribution policies were assessed for African countries using the policy information reported to WHO. A country was considered to have a policy for ANC and/or EPI distribution of LLINs if it declared the existence of a policy in its annual report to WHO on or before the year 2012. The report to WHO included both the existence of a policy and the year the policy was adopted. The delay in policy implementation was assessed by comparing the year of policy adoption to the 1st year in which the country reported distributing any LLINs through the relevant distribution channel.

The volume of LLINs distributed through ANC and EPI was assessed for countries in Africa, using the sum of LLINs distributed through all channels (campaigns, EPI, ANC, and other channels). The completeness of data available was assessed in terms of missing values and inconsistencies in reported totals. Annual reports that did not include specific information on the channel of distribution, or where the channel totals did not add up to within 10 % of the reported total nets distributed were excluded from the analysis. Countries were eligible for inclusion if they had reported complete distribution channel data in at least three of the 4 years from 2009 to 2012. If there were data for all four of these years, the most recent 3 years (2010–2012) were included. In all included countries, campaign nets were distributed in at least one of the included years. The most recent 3 years of data from each country were aggregated to account for year-to-year variation, especially due to campaigns.

The numbers of nets reportedly distributed through each distribution channel were summed over all included countries, to produce the proportion of the total nets reportedly distributed through each channel. An example of this calculation for ANC:

$$\frac{{\sum {\left( {\text{LLINs distributed via ANC}} \right)} }}{{\sum {\left( {\text{LLINs distributed via all channels}} \right)} }} = {\text{Proportion LLINs distributed via ANC}}$$

For countries reporting at least 3 years of LLINs distributed via ANC or EPI, an LLIN availability ratio was calculated. The LLIN availability ratio for a distribution channel represents the total number of nets reportedly distributed through the service relative to the total number of women or children attending that service. To assess whether the number of nets made available through ANC was sufficient to allow one LLIN to be given to every pregnant woman attending ANC, an ANC LLIN availability ratio pooling data across all included countries with LLIN distribution through ANC was calculated:

$$\frac{{\sum {\left( {\text{LLIN reported distributed via ANC}} \right)} }}{{\sum {\left( {\text{women reported attending ANC}} \right)} }} = {\text{ANC availability ratio}}$$

For the ANC availability ratio, both the denominator and numerator were reported by national malaria control programmes to the WHO Malaria Control Programme.

Similarly, for EPI, an LLIN availability ratio was calculated to assess whether the number of LLINs made available through this channel was sufficient for all the infants attending EPI services, pooling data across all included countries with LLIN distribution through EPI:

$$\frac{{\sum {\left( {\text{LLIN reported distributed via EPI}} \right)} }}{{\sum {\left( {\text{infants reported attending EPI}} \right)} }} = {\text{EPI availability ratio}}$$

In this case, the denominator (total infants attending EPI) was taken from a different source than the numerator: EPI coverage reports submitted to WHO by national EPI authorities. Because there is no clear consensus or recommendation as to the best age for LLIN distribution in EPI, two different LLIN availability ratios were calculated for EPI, using either the number of children who received DTP1 vaccination (normally at 6 weeks of age) or the number receiving measles vaccination (normally at 6 months of age).

$$\frac{{\sum {\left( {\text{LLIN reported distributed via EPI}} \right)} }}{{ \sum {\left( {\text{infants reported receieving DTP1}} \right)} }} = {\text{EPI }}\left( {\text{DTP1}} \right)\, {\text{availability ratio}}$$

and

$$\frac{{\sum {\left( {\text{LLIN reported distributed via EPI}} \right)} }}{{\sum {\left( {\text{infants reported receiving measles vaccine}} \right)} }} = {\text{EPI }}\left( {\text{Measles}} \right)\, {\text{availability ratio}}$$

In most countries, DTP1 coverage is higher than measles vaccination coverage, so the DTP1 vaccination comparison provides a more conservative estimate of LLIN availability.

## Results

Control programmes in malaria-endemic countries began reporting LLIN distribution data annually to WHO in 2008, and were asked at that time to provide historical data back to 2000. The review of data completeness revealed that no single African country reporting to WHO included complete distribution channel data for years before 2008. From 2008 onwards, most countries were reporting complete distribution channel data, but a large minority of reports each year still did not break down the data by distribution channel, up through the 2012 reports.

In 2012, of the 48 African countries reporting LLIN data to the WHO, 33 country programmes reported having a policy for LLIN distribution through ANC, and 25 reported having a policy for LLIN distribution through EPI. Of the 33 countries with a reported ANC distribution policy, one country had never reported implementation since policy adoption in 1998. Of the 25 countries with a reported EPI-based distribution policy, six countries had never reported implementation since policy adoption in years between 1998 and 2008. Furthermore, in Africa, seven countries reported distributing LLINs through ANC without having reported adopting an ANC LLIN distribution policy, and five countries reported distributing LLINs via EPI without having reported adopting an EPI LLIN distribution policy. ANC policies took an average of 2.3 years to be implemented (median: 2 years), while EPI policies took an average of 2.7 years to be implemented (median: 2 years). The range for this interval was wide, and in a few countries there were long delays: up to 11 and 9 years, for ANC and EPI, respectively.

In total, 38 country programmes were included in the analysis of continuous distribution (Table 1). In these countries (representing a population of approximately 805,404,900 people in 2012), 290,030,923 LLINs were distributed during the three-year window. Of these, 86 % were reportedly distributed via mass campaigns, 9 % via ANC, 4 % via EPI, and 2 % via other channels.

**Table 1:** Reporting country programmes data years included, total number of nets distributed and inclusion in different parts of continuous distribution channel analysis

Country programmes	Years included	Total nets	Distribution channel proportion analysis^a^	ANC availability ratio analysis^b^	EPI availability ratio analysis^c^
West					
Benin	09, 11, 12	6,720,585	Y	Y	Y
Burkina Faso	10, 11, 12	7,930,794	Y	Y	Y
Côte d’Ivoire	09, 10, 11	9,221,508	Y		Y
Gambia	09, 11, 12	1,182,883	Y	Y	Y
Ghana	10, 11, 12	13,042,900	Y		
Guinea-Bissau	10, 11, 12	1,179,669	Y	Y	Y
Liberia	09, 10, 11	2,474,400	Y		
Mali	10, 11, 12	7,128,578	Y	Y	Y
Nigeria	10, 11, 12	51,456,461	Y		
Senegal	09, 10, 11	5,342,486	Y		
Sierra Leone	10, 11, 12	3,598,535	Y	Y	Y
Togo	10, 11, 12	3,124,868	Y	Y	Y
Central					
Angola	10, 11, 12	3,876,147	Y	Y	Y
Cameroon	09, 10, 11	8,733,485	Y		
Central African Republic	09, 10, 12	1,078,274	Y		
Chad	09, 10, 11	3,909,081	Y		Y
Democratic Republic of Congo	10, 11, 12	32,952,748	Y	Y	Y
Equatorial Guinea	09, 11, 12	19,035	Y	Y	
Sao Tome and Principe	10, 11, 12	157,700	Y	Y	
South					
Botswana	10, 11, 12	148,500	Y		
Mozambique	10, 11, 12	7,439,387	Y	Y	
Swaziland	10, 11, 12	159,805	Y		
East					
Burundi	10, 11, 12	4,751,975	Y		Y
Comoros	10, 11, 12	270,120	Y		
Djibouti	10, 11, 12	54,800	Y		
Eritrea	10, 11, 12	1,179,640	Y		
Ethiopia	10, 11, 12	24,337,326	Y		
Kenya	10, 11, 12	14,461,002	Y	Y	Y
Madagascar	10, 11, 12	9,436,883	Y	Y	Y
Malawi	10, 11, 12	9,289,178	Y	Y	
Rwanda	10, 11, 12	7,255,887	Y		Y
Somalia	10, 11, 12	796,698	Y		
Sudan	10, 11, 12	3,692,659	Y		
Tanzania (mainland)	10, 11, 12	24,573,301	Y	Y	Y
Tanzania (Zanzibar)	09, 10, 11	348,250	Y		
Uganda	10, 11, 12	9,109,747	Y		
Zambia	10, 11, 12	7,278,762	Y	Y	
Zimbabwe	09, 10, 12	2,316,866	Y		
*Total: 38*		*290,030,923*	*38*	*17*	*16*

Data source: total nets distributed annually, reported by national malaria control programmes to the WHO Global Malaria Programme

^a^Countries included in the analysis of the proportion of total nets distributed through each channel

^b^Countries included in the analysis of ANC availability ratio: the number of nets reportedly distributed via ANC over the number of women reportedly attending ANC services

^c^Countries included in the analysis of the EPI availability ratio: the number of nets reportedly distributed via EPI over the number of children reportedly attending EPI services

Seventeen countries had sufficient data to be included in the ANC availability ratio calculation, and 16 had sufficient data to be included in the EPI availability calculation (Table 1). The ANC LLIN availability ratio, in countries with active distribution for this channel, was 55 % (Table 2). Thus, LLINs reportedly distributed via ANC were sufficient to provide one LLIN to 55 % of the women reportedly attending this service. The LLIN availability ratio for EPI was calculated using both DTP1 and measles visits as denominators. With the DTP1 visits as denominator, the availability ratio for countries actively distributing LLINs through EPI was 34 % (Table 2). With measles vaccination visits, the availability ratio was 37 % (Table 2). Thus, LLINs reportedly distributed via EPI were sufficient to provide one LLIN to 34–37 % of the infants attending this service. When availability ratios were calculated for individual countries, performance varied greatly for both ANC and EPI distribution, with the lowest availability ratios below 10 %, and the highest greater than 90 %.

**Table 2:** Comparison of ANC and EPI-based LLIN distribution in African countries

	ANC	EPI	
Policy and implementation			
Countries with a reported distribution policy	33	25	
Average years between policy and implementation	2.3	2.7	
Proportion of LLINs distributed via each channel			
Proportion of total LLINs distributed through the channel (%)	9	4	

Distribution channel availability ratio	ANC (%)	DTP1 (%)	Measles (%)
Availability ratio	55	34^b^	37^c^
Missed opportunities through channel^a^	45	66^b^	63^c^

^a^The proportion of reported women attending ANC or children attending EPI for whom an LLIN was not available

^b^Ratio calculated using the number of children who received DTP 1 vaccination as the denominator

^c^Ratio calculated using the number of children who received Measles vaccination as the denominator

## Discussion

**Fig. 1:**
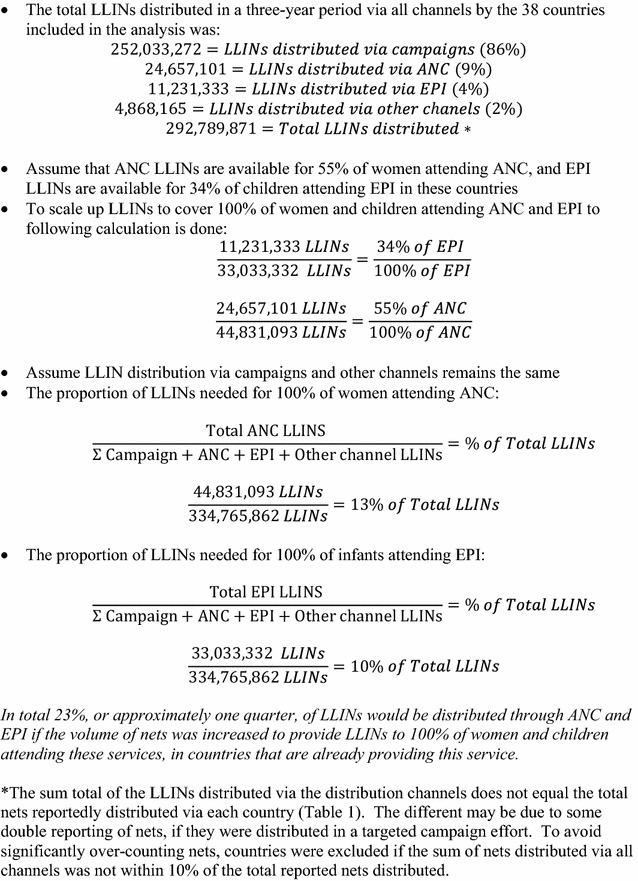
Scale-up of LLIN distribution—calculation based on previous distribution

**Fig. 2:**
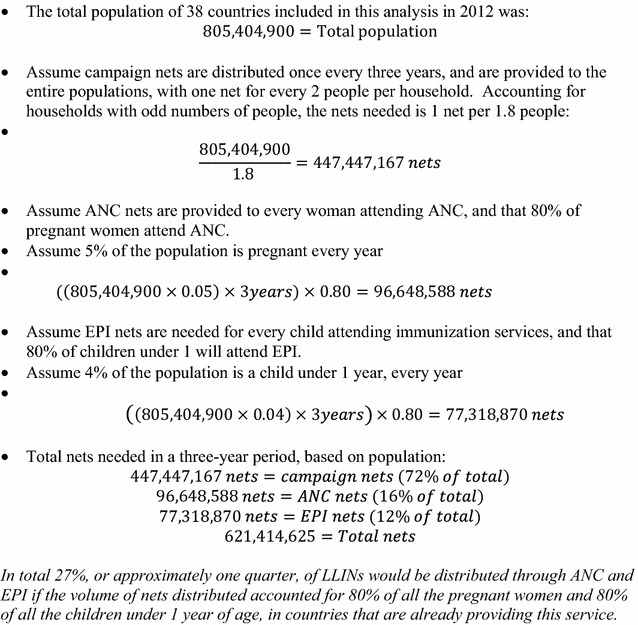
Scale-up of LLIN distribution—calculation based on population

These availability ratios suggest that in countries where LLIN distribution was occurring through ANC and EPI, the number of LLINs distributed to ANC and EPI clinics was not enough to allow every woman or child attending these services to receive an LLIN (as recommended by WHO). Consequently, nearly half of women attending ANC and more than 60 % of infants attending EPI represent a missed opportunity to distribute an LLIN to a pregnant woman or child possibly in need of one (Table 2). This suggests that both ANC and EPI visits are under-utilized for distribution of LLINs by national malaria programmes and international funding agencies, whether estimated using distribution data (Fig. 1) or population data (Fig. 2). It is worth noting that the policies in place may not specify that LLINs should be given to all women and children attending ANC and EPI, as stated in the WHO recommendation [4].

This shortfall in LLINs for continuous distribution through ANC and EPI could be due to under-allocation of LLINs from national central supplies to facilities for use in these channels. Webster and colleagues identify an inconsistent supply of LLINs at facilities as a barrier to continuous distribution through ANC [11] and Theiss-Nyland and colleagues identified frequent stock-outs and stock shortages of LLINs intended for ANC and EPI based distribution [12]. However, LLINs are often allocated towards a specific distribution channel before arriving in country, based on funding allocated to different distribution methods. This shortfall in meeting the needs of these distribution programmes is likely due to inadequate supply of LLINs to countries (Figs. 1, 2). By these calculations, more than 40 million additional nets, or approximately one quarter of the total nets distributed, may be needed to meet the demand of ANC and EPI programmes (Figs. 1, 2). The Global Fund to Fight AIDS, Tuberculosis and Malaria (GF) includes “[the] number of long-lasting insecticidal nets distributed to targeted risk groups through continuous distribution” as a core indicator for malaria programme monitoring [13]. However, this target does not require a population-based denominator [13]. This number is compared to the target set by the programme [13]. As a result, countries are not required to compare the number of LLINs provided against the populations attending continuous distribution services (as has been done in this paper). If the indicators recommended by key funding institutions measure only whether or not there is provision of LLINs to pregnant women and infants, and not the completeness of that provision, country programmes may have little financial incentive to monitor and improve the adequacy and effectiveness of the supply of nets.

While it appears that ANC-based continuous LLIN distribution has been more effectively implemented than EPI-based distribution, national programmes only reported distributing enough LLINs through ANC for half the number of women attending ANC at least once. The higher availability ratio seen in ANC-based distribution may be the result of greater emphasis and focus on this channel, as part of broader efforts to combat the effects of malaria in pregnancy. While many studies have investigated ANC-based distribution [11, 14–17], only one pilot programme was found that focused on EPI-based LLIN distribution [18]. Under-utilization of these continuous distribution channels could be one factor preventing programmes from achieving or maintaining universal LLIN coverage.

These findings also suggest that, despite WHO recommendations for continuous distribution, most countries are still relying heavily on mass distribution campaigns to distribute LLINs. These campaigns are often still necessary for increasing national LLIN ownership and maintaining coverage, but pregnancies and births that occur between campaigns represent vulnerable populations potentially unprotected without effective continuous distribution programmes. Likewise, campaign nets that degrade over time need to be replenished through continuous distribution channels in order to maintain high coverage.

The dataset used to make these comparisons presents a number of limitations. The quantities of LLINs distributed, and the number of women and children attending ANC and EPI services are assumed to be the best estimates from service delivery records and surveys in each country and year included. These are the only data available at a national level on the total nets distributed via different channels. However, there were too many missing data points for LLIN quantities in the dataset before the year of 2009, which limited the analysis to 3 years, and made historical comparisons before continuous distribution recommendations impossible. The EPI attendance numbers were provided by WHO EPI nationally reported vaccination coverage, and survey data [19]. The ANC numbers, by comparison, were reported by the national malaria control programmes from each country, along with the LLIN information. Although 30 countries reported distributing LLINs via ANC in the years included, only 17 of those countries provided ANC attendance numbers, limiting the analysis. A possible bias could be that only countries with better ANC distribution performance provided ANC attendance numbers. This means that the difference between ANC and EPI LLIN availability ratios may be a result of bias, rather than a true difference. The years of data included were not the same in all the countries, and eight out of the total 38 countries included in the analysis did not have 3 consecutive years of data due to incomplete reporting in some years (Table 1). The LLIN quantities reported likely represent the number of nets distributed from central storage to facilities in each country, while the number of ANC and EPI visits come from reported service delivery. Finally, only data up to 2012 were analysed; more recent data may show improvements in distribution of LLINs through these channels.

Despite these limitations, this analysis is still useful to paint a broad picture of continuous distribution through ANC and EPI. While studies have modelled the potential coverage and the cost effectiveness of continuous distribution [14, 20–22], few studies have critically evaluated the extent to which continuous distribution is serving its target population [11].

Beyond direct programme performance, this study also identified a deficiency in ANC and EPI-based continuous distribution programme monitoring and evaluation. Malaria programmes have relied on household surveys to monitor and assess the ownership and coverage of LLINs within a country. Surveys are arguably the best way to assess the outcome measures of LLIN ownership and use, but in the past most household surveys did not collect information on the source LLIN, making analyses like this very difficult. The most recent Demographic and Health Surveys 7 (DHS-7) questionnaires does include two questions intended to identify both the programme source and point of distribution for LLINs located in homes [23]. Unfortunately, the programme source of LLINs has yet to appear in the coded datasets from DHS-7 surveys in Africa that are available for analysis [23]. While information on the source of LLINs is a welcome addition to household survey data, data available from facilities and programmes, such as LLIN availability at the health facility and the proportion of women and infants who actually receive an LLIN out of those eligible, provide further insight about the process measures associated with programme performance.

Country malaria control programmes can adopt monitoring tools like these, which can serve as benchmarks for direct programme performance, and provide insight into areas of improvement for country programmes. Given the integrated nature of continuous distribution via ANC and EPI, malaria programmes may be able to gain from both the experience of, and the systems put in place by, EPI and ANC in each country. By building routine data collection and reporting systems for LLIN service delivery, malaria programmes can monitor their performance against ANC and EPI, using routine health facility data, and identify areas that can serve as examples of best practices, and areas where more resources and support are needed.

## Conclusions

These continuous LLIN distribution channels appear to be under-utilized, especially EPI-based distribution. This analysis illustrates the need to strengthen both the continuous distribution of LLINs, as well as the data collection and reporting systems necessary to effectively monitor a routine programme of this nature. However, quality data from more countries are needed for consistent and reliable programme performance monitoring. A greater focus on routine data collection, monitoring and reporting on LLINs distributed through both ANC and EPI can provide insight into both strengths and weaknesses of continuous distribution, and improve the effectiveness of these delivery channels. By building on the ANC and EPI service registers in use in countries, malaria programmes and international partners supporting these programmes, can take advantage of existing routine data structure to monitor programme performance. For integrated malaria programmes of this nature, sharing data with ANC and EPI programmes can also provide valuable estimations of target populations that can be reached through these channels. ANC and EPI services provide an important opportunity for LLIN distribution programmes to reach biologically vulnerable women and children, and fill gaps in population coverage. In order to take advantage of these distribution points, LLINs need to be made available, consistently, for all women and children attending these services.
